# Associations of Despair With Suicidality and Substance Misuse Among Young Adults

**DOI:** 10.1001/jamanetworkopen.2020.8627

**Published:** 2020-06-23

**Authors:** William E. Copeland, Lauren Gaydosh, Sherika N. Hill, Jennifer Godwin, Kathleen Mullan Harris, E. Jane Costello, Lilly Shanahan

**Affiliations:** 1 Vermont Center for Children, Youth, and Families, Department of Psychiatry, University of Vermont College of Medicine, Burlington; 2 Public Policy Studies, Center for Medicine, Health, and Society, Vanderbilt University, Nashville, Tennessee; 3 Department of Psychiatry, Duke University Medical Center, Durham, North Carolina; 4 Center for Child and Family Policy, Duke University, Durham, North Carolina; 5 Carolina Population Center, Department of Sociology, University of North Carolina at Chapel Hill; 6 Department of Psychiatry and Behavioral Sciences, Duke University Medical Center, Durham, North Carolina; 7 Jacobs Center for Productive Youth Development, Department of Psychology, University of Zürich, Zürich, Switzerland

## Abstract

**Importance:**

*Deaths of despair* is a term that has recently been used to describe the increases in premature mortality from suicides, drug overdoses (particularly from opiates), and alcohol-related liver disease among US adults. Despite the use of the term *despair*, its role in these causes of premature death has not been empirically tested.

**Objective:**

To test whether despair among young adults is associated with suicidal thoughts and behavior, alcohol misuse, and drug misuse.

**Design, Setting, and Participants:**

The Great Smoky Mountains Study is a Southeastern, mixed urban-rural population-based cohort study conducted from November 10, 1992, to September 22, 2015. A total of 1420 participants originally 9, 11, and 13 years of age were followed up 11 times to 30 years of age (11 230 person-observations). A total of 1154 of 1400 living participants (82.4%) were assessed at 30 years of age. Statistical analysis was performed from May 7, 2019, to April 10, 2020.

**Exposures:**

Participants were assessed with structured interviews for indicators of despair (eg, hopelessness, helplessness, low self-worth, and feeling unloved). Despair was assessed with items from structured interviews: the Child and Adolescent Psychiatric Assessment and the Young Adult Psychiatric Assessment.

**Main Outcomes and Measures:**

Structured interviews were used to assess suicidal thoughts and behavior, substance use, and *Diagnostic and Statistical Manual of Mental Disorders* (Fifth Edition) alcohol use disorder and drug use disorder (including opioids) in young adulthood (2424 observations of 1266 individuals between 25 and 30 years of age).

**Results:**

This study included 1420 individuals (790 male individuals). During young adulthood (25 and 30 years of age), the 3-month weighted prevalence of any despair was 19.5% (476 of 2424 observations) with 7.6% of participants (201 of 2424 observations) reporting 2 or more despair items. In longitudinal, lagged models, despair scores (range, 0-3) were associated with more suicidal thoughts and behaviors (odds ratio [OR], 1.5; 95% CI, 1.1-2.0), illicit drug use (OR, 1.7; 95% CI, 1.2-2.5), and opioid use (OR, 1.9; 95% CI, 1.1-3.3) but not alcohol use disorder (OR, 0.8; 95% CI, 0.6-1.2). These associations persisted after accounting for sociodemographic factors (eg, poverty and educational level), lagged outcome status, and lagged depression status. The associations between despair and study outcomes were stronger in models accounting for long-term measures of despair extending back to childhood. There was no consistent pattern of moderation by sociodemographic factors.

**Conclusions and Relevance:**

This study’s findings suggest an empirical basis for longitudinal associations between despair and several, but not all, precursors of “deaths of despair” in rural Appalachia. Individual despair should be studied as a potential factor associated with morbidity and impairment in young adulthood.

## Key Points

**Question:**

Is despair associated with drug or alcohol misuse or suicidal thoughts and behaviors among young adults?

**Findings:**

In this population-based cohort study in rural Appalachia, despair was longitudinally associated with higher rates of suicidal thoughts and behavior, illicit drug use, and opioid use, even after adjusting for sociodemographic factors, prior outcome status, and prior depressive disorder status; despair was not associated with alcohol use disorder. There was no consistent pattern of moderation by race/ethnicity, poverty status, sex, or educational level.

**Meaning:**

Despair early in life is longitudinally associated with several (but not all) putative despair-related diseases.

## Teaser

This cohort study uses data from the Great Smoky Mountains Study to examine whether despair among young adults is associated with suicidal thoughts and behavior, alcohol misuse, and drug misuse.

## Introduction

In recent decades, income stagnation and economic declines have coincided with premature deaths from suicide, alcoholism, and drug (especially opioid) overdoses, particularly among white non-Hispanic adults with low education in rural areas.^1^ Recent work reports that premature mortality from these causes occurs not only among middle-aged white adults but also among young adults and additional racial/ethnic groups.^2^ This reversal of US life-expectancy improvements was coined *deaths of despair*^1,3,4,5^ and has raised public and scientific interest. Studies on deaths of despair have not measured the central empirical claim that despair is a shared risk pathway to suicides and alcohol- and drug-related deaths or their precursors: suicidal behaviors and thoughts, alcohol misuse and dependence, and illicit drug use (ie, the “diseases of despair”).^6^ To our knowledge, to date, despair has not been studied as an independent construct in and of itself.

Despair is defined as “a state of mind in which there is an entire want of hope.”^7^ From a psychological perspective, this definition focuses on a cognitive state that includes defeat, guilt, worthlessness, learned helplessness, pessimism, and limited positive expectations for the future. Some of these cognitions overlap with specific criteria for *Diagnostic and Statistical Manual of Mental Disorders* Fifth Edition (*DSM-5*)–based depressive disorders (eg, feeling worthless and major depressive disorder), whereas others do not (eg, helplessness and loneliness). The present study tests the hypothesis that despair is a shared risk pathway to diseases of despair. We use data from a prospective, longitudinal, community-representative study of more than 25 years conducted in a rural area of the Southeastern United States. The sample was recruited from parts of the Appalachian region, which has had particularly high levels of premature mortality and deaths of despair, including from opiate overdoses.^8,9^ This analysis aims to test whether despair early in life might play a role in the premature mortality crisis.

We first examined the prevalence of despair from childhood to midlife. Next, we tested whether despair is associated with suicidal thoughts and behaviors, alcohol misuse, and drug misuse in young adulthood (between 25 and 30 years of age). Third, we used longitudinal lagged analyses to test directionality between despair and its hypothesized diseases over time. That is, does despair precede the diseases of despair, do the diseases precede despair, or both? We hypothesize that despair will be associated with higher levels of diseases of despair but do not have specific hypotheses for which diseases would have strongest associations. To truly illuminate the role of despair, we tested whether associations of despair with outcomes are independent of *DSM*-based depressive disorders with which despair is commonly associated. Finally, we tested whether associations are particularly strong in certain demographic groups, including those with low educational levels and those from a white non-Hispanic background.

## Methods

### Participants

The Great Smoky Mountains Study is a representative cohort study of children in 11 mostly rural counties of North Carolina.^10^ Three cohorts of children, aged 9, 11, and 13 years, were recruited from a pool of approximately 12 000 children using a 2-stage sampling design, resulting in 1420 participants (630 girls).^10^ First, potential participants were randomly selected from the population using a household equal probability design. Next, participants were screened for risk of psychopathological conditions; participants whose screening results indicated a high risk were oversampled in addition to a random sample of the rest of the participants. In addition, Native American participants were oversampled to constitute 25% of the sample. In all statistical analyses, sampling weights are applied to adjust for the differential probability of selection and to allow results to generalize to the broader population of children from which the sample was drawn (eFigure in the Supplement).^10,11,12^ Before all interviews, parents and children signed informed consent or assent forms. The study protocol and consent forms for each assessment were approved by the Duke University Medical Center institutional review board. Participants received payment for their time ($100 for the most recent wave). This study followed the Strengthening the Reporting of Observational Studies in Epidemiology (STROBE) reporting guidelines.^13^

Annual assessments of the 1420 children were completed during the period from 9 to 16 years of age (6674 total observations; 1993-2000) and then again at 19, 21, 25, and 30 years of age (4556 observations of 1336 participants; 1999-2015), for a total of 11 230 total assessments. Interviews were completed separately by a parent figure and the participant until 16 years of age and by the participant only thereafter.

### Measures

Despair was assessed with items from structured interviews: the Child and Adolescent Psychiatric Assessment^14,15^ and the Young Adult Psychiatric Assessment (YAPA).^16^ These structured interviews assess the psychiatric symptoms needed to make diagnoses but also a broad range of associated constructs. To derive a scale of despair, all interview items were reviewed for consistency with how despair has been defined in recent publications on deaths of despair.^6,17^ Seven items of the Child and Adolescent Psychiatric Assessment and YAPA interviews capture the cognitive features of despair: loneliness, hopelessness, feeling unloved, helplessness, low self-esteem, frequent worries, and feeling sorry for oneself. eTable 1 in the Supplement includes a full list of despair-related items, their operational definitions, and their overlap with *DSM* constructs. Two items used in the despair scale are criteria for *DSM*-defined persistent depressive disorder or dysthymia (ie, a pattern of dysphoria lasting more than 2 years), 1 item is a criterion for major depressive disorder, 1 item is a criterion for generalized anxiety disorder, and 3 items are not criteria for any psychiatric disorder. The 7 dichotomous despair indicators were summed into a despair score. The internal consistency of this scale was acceptable (Cronbach α = .73). This scale was winsorized at 3 or more indicators of despair to aid in the presentation of results. Despair may also occur in the emotional (eg, sadness and anhedonia), behavioral (eg, reckless and high-risk behaviors), and biological (eg, signs of biological depletion such as high allostatic load) domains, but these domains were not the focus of the present study.^6^

### Diseases Associated With Dispair

The 3 diseases of despair assessed in this study included suicidal thoughts and behaviors, alcoholism, and drug misuse, including opioid use. All outcomes were assessed using the YAPA,^16^ which focuses on the 3 months immediately preceding the interview to minimize forgetting and recall bias. Additional sociodemographic variables were collected on sex, race/ethnicity, educational level, and falling below the federal poverty thresholds based on income and family size.

#### Suicidal Thoughts and Behaviors

eTable 2 in the Supplement provides definitions for passive suicidal ideation, active suicidal ideation, suicide plans, or suicide attempts. The definitions of these constructs are consistent with the Columbia Classification Algorithm of Suicide Assessment.^18^ Participants were considered positive for suicidal thoughts and behaviors if they reported any of these.

#### Alcohol and Drug Misuse

The substance use section of the YAPA first asks about frequency of use for specific substances, followed by a detailed section on symptoms and impairment; questions in this section were asked only if substance use was reported. The module assessed symptoms of *DSM-5* substance use disorder.^19^ A 2-week test-retest study to determine the reliability of participant reporting for the number of substance misuse or dependence symptoms had an intraclass correlation coefficient of 0.98.^15^

For the present analysis, the primary outcome variable for alcohol misuse was meeting full diagnostic criteria for *DSM-5* alcohol use disorder (ie, a well-known precursor of liver cirrhosis). The following illicit drugs were assessed for this study: cocaine, crack, amphetamines, methamphetamine, inhalants, nitrite inhalants, ecstasy, heroin, other opioids, oxycodone (could include lawful use), LSD (lysergic acid diethylamide), PCP (phencyclidine), psilocybin, and sedatives. We assessed any illicit drug use meeting the criteria for an illicit drug use disorder, as well as use of any opioids only (ie, oxycodone, other opioids, or heroin). Cannabis was not included in the illicit drug variable because cannabis-related overdoses are not associated with death and have not been studied in the literature on deaths of despair.

### Statistical Analysis

Statistical analysis was performed from May 7, 2019, to April 10, 2020. All statistical analyses accounted for the 2-stage sampling design using sampling weights. Each participant was assigned a weight inversely proportional to their probability of selection. All models also used the generalized estimating equations option within SAS PROC GENMOD (SAS Institute Inc) to derive robust variance (sandwich-type) estimates to adjust SEs for the stratified design and multiple observations for each participant. Associations between variables were tested using weighted logistic regression models (for binary outcomes such as suicidal behavior or meeting a *DSM* diagnosis) and ordered multinomial regression models (for number of despair indicators).

Longitudinal analyses focused on 2424 observations of 1266 individuals between 25 and 30 years of age. In longitudinal models, the given outcome (eg, time 2 suicidality) was regressed on both the lagged values of the outcome variable (time 1 suicidality) and the independent variable (time 1 despair score). Consistent with common conventions, all percentages provided in the results are weighted, and sample sizes are unweighted. All *P* values were from 2-sided tests and results were deemed statistically significant at *P* < .05.

### Missing Data

Across all assessments, 83.0% of possible interviews (11 230 of 13 529) were completed. Of the 1420 original participants, 1266 (89.2%) were followed up at least once in young adulthood at 25 or 30 years of age. Despair at earlier assessments was not associated with lower levels of participation in young adulthood. Missing individual despair items within completed young adult interviews were rare (approximately 1%). For generalized linear models, PROC GENMOD excludes any observation with a missing value for any variable involved in the model (approximately 5% of observations).

## Results

### Prevalence of Despair and Associations With Sociodemographic Factors

The Great Smoky Mountains Study includes 1420 participants (790 male and 630 female participants). The Figure shows the percentage of participants reporting individual indicators of despair and any despair from 9 to 30 years of age. The prevalence of despair was between 5% and 1% in childhood and adolescence (9-21 years) but increased to around 20% in young adulthood (between 25 and 30 years). Levels of any despair and total despair scores increased by age. Each of the individual indicators of despair followed this developmental pattern of increasing with age.

**Figure.**
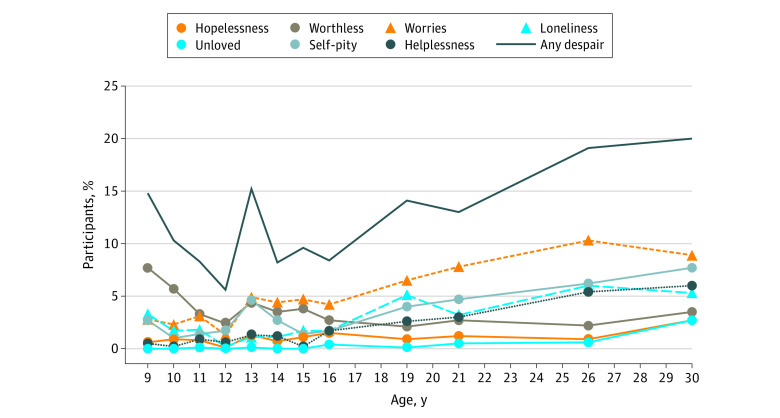
Percentage of Participants Reporting Individual Indicators of Despair and Any Despair by Age Analysis is based on 11 230 observations of 1420 participants.

Next, we tested the associations between despair and sociodemographic factors in young adulthood (between 25 and 30 years of age; 2424 observations of 1266 individuals). Table 1 shows the 3-month prevalence of counts on the despair summary scale in young adulthood and associations with sociodemographic factors. The 3-month weighted prevalence of any despair was 19.5% (476 of 2424 observations) and was 7.6% (201 of 2424 observations) for reporting 2 or more items. Despair scores did not differ between male and female participants. Levels of despair differed by race/ethnicity; compared with white participants, African American participants reported much higher rates of despair and Native American participants reported lower levels of despair. Lower educational level and falling below the federal poverty line were associated with higher levels of despair. All sociodemographic factors were subsequently used as covariates in analyses testing associations with the diseases of despair.

**Table 1.** Prevalence of Counts on the Despair Summary Scale in Young Adulthood (at 25 and 30 Years of Age) and Associations of Summary Score With Young Adult Demographic Variables^a^

Variable	Despair score, No. (%)^b^				*P* value^c^
	Score of 0	Score of 1	Score of 2	Score of ≥3	
Total	1937 (80.5)	275 (12.0)	94 (4.1)	107 (3.5)	[Reference]
Sex					
Female	893 (77.7)	144 (13.7)	54 (5.4)	54 (3.2)	[Reference]
Male	1044 (83.5)	131 (10.2)	40 (2.6)	53 (3.8)	.20
Race/ethnicity					
White	1288 (81.6)	217 (11.4)	72 (3.8)	93 (3.3)	[Reference]
African American	106 (63.1)	27 (21.9)	13 (8.2)	8 (6.9)	<.001^d^
Native American	543 (92.2)	31 (5.3)	9 (1.5)	6 (1.0)	<.001^d^
Educational level					
No high school degree	249 (69.8)	42 (17.5)	17 (7.5)	20 (5.2)	.002^d^
High school only	418 (76.1)	67 (15.1)	14 (2.6)	31 (6.2)	.04^d^
Some college	642 (80.6)	94 (9.2)	45 (5.6)	46 (4.6)	.04^d^
4-y Degree	519 (83.0)	72 (13.0)	18 (3.0)	10 (1.1)	[Reference]
Poverty					
Yes	353 (64.6)	96 (18.9)	29 (6.3)	56 (10.2)	[Reference]
No	1449 (83.7)	170 (10.6)	64 (3.6)	50 (2.0)	<.001^d^

^a^ Based on 2424 observations of 266 individuals assessed at 25 and 30 years of age.

^b^ All percentages are weighted, and all numbers are unweighted.

^c^ Representing results from ordered multinomial regression regressing despair scores on young adult demographic variables.

^d^ Significant at *P* < .05.

Despair was associated with a diagnosis of a *DSM* depressive disorder (odds ratio [OR], 3.8; 95% CI, 2.9-4.9; *P* < .001). Of the 476 young adult observations in which at least 1 symptom of despair was reported, however, 94 (19.7%) of them met criteria for a *DSM* depressive disorder, suggesting that despair encompasses a much broader group than those with depression.

### Associations With Suicidal Thoughts and Behaviors, Alcohol Misuse, and Illicit Drug Misuse

Table 2 shows concurrent associations between despair scores in young adulthood and putative outcomes adjusted for sociodemographic factors. Higher despair scores were associated with higher levels of suicidal thoughts and behavior, illicit drug use, illicit drug disorders, opioid use, and any disease of despair. Despair scores were not significantly associated with alcohol use disorder between 25 and 30 years of age.

**Table 2.** Associations of Despair Score With Young Adult (at 25 and 30 Years of Age) Outcomes at the Same Observation^a^

Outcome	Participants, No. (%)^b^					*P* value^c^
	Total	Despair scores				
		0	1	2	3+	
Total	2424 (100)	1937 (80.5)	275 (12.0)	94 (4.1)	107 (3.5)	NA
Suicide						
Yes	160 (5.9)	83 (3.6)	29 (9.7)	17 (13.1)	31 (27.9)	<.001^d^
No	2253 (94.1)	1854 (96.4)	246 (90.3)	77 (86.9)	76 (72.1)	
Alcohol use disorder						
Yes	132 (7.6)	99 (6.7)	18 (10.7)	8 (9.8)	7 (3.4)	.48
No	2292 (92.4)	1838 (93.3)	257 (89.3)	86 (90.2)	100 (96.6)	
Illicit drug use						
Yes	137 (4.7)	85 (3.7)	24 (5.6)	8 (6.8)	20 (18.5)	.04^d^
No	2247 (95.3)	1824 (96.3)	250 (94.4)	86 (93.2)	87 (81.5)	
Illicit drug use disorder						
Yes	88 (2.9)	46 (1.4)	16 (5.6)	7 (6.0)	19 (17.6)	<.001^d^
No	2336 (97.1)	1891 (98.6)	259 (94.4)	87 (94.0)	88 (82.4)	
Opioid use						
Yes	55 (2.2)	31 (1.6)	8 (1.0)	6 (5.9)	10 (13.6)	.007^d^
No	2328 (97.8)	1877 (98.4)	266 (99.0)	88 (94.1)	97 (86.4)	

Abbreviation: NA, not applicable.

^a^ Based on 2424 observations of 1266 individuals.

^b^ All percentages are weighted, and all numbers are unweighted. Covariates included sex, race/ethnicity, educational level, and poverty.

^c^ Representing results from logistic models regressing young adult outcome variables on concurrent despair scores.

^d^ Significant at *P* < .05.

Bidirectional temporal associations between despair and outcomes were tested using a series of longitudinal lagged models (Table 3). Outcome status at 25 or 30 years of age was regressed on status of variables at the prior wave (eg, suicidal thoughts and behavior at 30 years regressed on despair score at 25 years). Models were adjusted for status of the outcome variable at the prior wave (eg, suicidal thoughts and behavior at 25 years). Because each lagged model is based on 2 time points, each participant could contribute up to 2 observations to these analyses. Models were performed using generalized estimating equations to account for repeated observations. All sociodemographic covariates were included.

**Table 3.** Longitudinal Models Between Outcomes at 25 and 30 Years of Age and Variables at 21 and 25 Years of Age Adjusted for Sociodemographic Covariates and Depression Status^a^

Outcome	Lagged variable	Adjusted for sociodemographic covariates		Adjusted for sociodemographic covariates and lagged depression status	
		OR or β (95% CI)	*P* value	OR or β (95% CI)	*P* value
Logistic regression					
Suicidal thoughts or behaviors	Despair	1.5 (1.1 to 2.0)	.02^b^	1.5 (1.1 to 2.1)	.02^b^
Alcohol use disorder	Despair	0.8 (0.6 to 1.2)	.34	0.8 (0.5 to 1.2)	.31
Illicit drug use	Despair	1.7 (1.2 to 2.5)	.006^b^	1.7 (1.1 to 2.8)	.02^b^
Illicit drug use disorder	Despair	1.0 (0.6 to 1.7)	.99	0.8 (0.5 to 1.2)	.26
Opioid use	Despair	1.9 (1.1 to 3.3)	.02^b^	1.9 (1.0 to 3.5)	.04^b^
Ordered multinomial models					
Despair	Suicidal thoughts or behaviors	−0.3 (−1.1 to 0.5)	.43	−0.7 (−1.4 to −0.1)	.05^b^
Despair	Alcohol disorder	0.1 (−0.4 to 0.6)	.71	0.1 (−0.7 to 0.5)	.67
Despair	Illicit drug use	0.1 (−0.4 to 0.6)	.71	0.1 (−0.5 to 0.6)	.83
Despair	Illicit drug disorder	−0.1 (−0.7 to 0.5)	.81	−0.1 (−0.6 to 0.5)	.83
Despair	Opioid use	−0.3 (−1.0 to 0.4)	.40	−0.4 (−1.1 to 0.3)	.25

Abbreviation: OR, odds ratio.

^a^ Based on 2424 observations of 1266 individuals. The top 5 rows represent results from logistic regression regressing young adult outcome variables on lagged despair scores. The bottom 5 rows represent results from ordered multinomial models regressing despair scores on lagged young suicidality and substance use variables. Sociodemographic covariates included sex, race/ethnicity, educational level, poverty, and lagged value of the outcome variable.

^b^ Significant at *P* < .05.

The first 5 rows of results in Table 3 display logistic regression models estimating young adult outcomes (between 25 and 30 years of age; 2424 observations of 1266 individuals) from previous despair levels. Prior despair was associated with elevated levels of suicidal thoughts and behaviors (OR, 1.5; 95% CI, 1.1-2.0), illicit drug use (OR, 1.7; 95% CI, 1.2-2.5), and opioid use (OR, 1.9; 95% CI, 1.1-3.3) but not with alcohol use disorder (OR, 0.8; 95% CI, 0.6-1.2) or illicit drug disorder (OR, 1.0; 95% CI 0.6-1.7) (Table 3 and eTable 3 in the Supplement). The ORs represent the change in odds of the outcome given a 1-unit increase in the despair score (range, 0-3). In analyses assessing the difference between different despair scores, risk tended to increase with the number of despair items reported, with evidence that 2 or more items were associated with moderate to large effects (ORs >2; eTable 4 in the Supplement). Finally, the adjusted lagged models were performed again, adjusted for lagged depressive disorder status (in addition to other covariates). The aim was to test whether the lagged association of despair was accounted for by depression or whether despair was independently associated with young adult outcomes. In the model adjusted for depressive disorder (Table 3), lagged despair was still associated with suicidal thoughts and behaviors (OR, 1.5; 95% CI, 1.1-2.1), illicit drug use (OR, 1.7; 95% CI, 1.1-2.8), and opioid use (OR, 1.9; 95% CI, 1.0-3.5).

The final 5 rows of Table 3 show results from ordered multinomial regression models testing the reverse pattern of prior status of suicidal thoughts and behaviors and substance use variables estimating current despair scores. None of these diseases were associated with later despair scores. All models in Table 3 were performed again, with 3 variations: (1) using a nonwinsorized despair variable (ie, full range from 0 to 7), (2) using a dichotomous despair variable for the presence or absence of any despair indicators, and (3) using 7 despair sum scores that systematically left out 1 of the indicators. In all cases, the pattern of results was similar to those reported in Table 3. These results are available on request from the first author.

### Long-term Despair Exposure

Table 4 shows 2 alternative definitions of long-term despair exposure: (1) total despair scores across all prior observations (cumulative despair levels) and (2) total observations reporting any despair (cumulative despair observations). Each measure was averaged over the total number of available observations for that individual. Thus, the range for cumulative despair levels was from 0 to 4.5, and the range for cumulative despair observations was from 0 to 1. Both measures of long-term despair were associated with increased risk for suicide (cumulative despair levels: OR, 2.6; 95% CI, 1.5-4.7; cumulative despair observations: OR, 10.7; 95% CI, 3.5-32.9), illicit drug use (cumulative despair levels: OR, 2.5; 95% CI, 1.3-5.0; cumulative despair observations: OR, 6.0; 95% CI, 1.6-22.9), illicit drug use disorder (cumulative despair levels: OR, 2.3; 95% CI, 1.3-4.4; cumulative despair observations: OR, 9.4; 95% CI, 2.4-36.7), and opioid use (cumulative despair levels: OR, 3.3; 95% CI, 1.4-7.7; cumulative despair observations: OR, 7.5; 95% CI, 0.9-65.5). In all cases, the associations were stronger than those observed in the lagged models, suggesting the importance of indexing long-term despair exposure.

**Table 4.** Longitudinal Models Between Different Definitions of Long-term Despair Exposure and Young Adult Outcome (at 25 and 30 Years of Age), Adjusted for Covariates^a^

Outcome	Cumulative despair levels		Cumulative despair observations	
	OR (95% CI)	*P* value	OR (95% CI)	*P* value
Suicidal thoughts or behaviors	2.6 (1.5-4.7)	<.001^b^	10.7 (3.5-32.9)	<.001^b^
Alcohol use disorder	0.9 (0.5-1.7)	.75	0.9 (0.2-3.7)	.84
Illicit drug use	2.5 (1.3-5.0)	.008^b^	6.0 (1.6-22.9)	.008^b^
Illicit drug use disorder	2.3 (1.3-4.4)	.008^b^	9.4 (2.4-36.7)	.001^b^
Opioid use	3.3 (1.4-7.7)	.007^b^	7.5 (0.9-65.5)	.07^b^

Abbreviation: OR, odds ratio.

^a^ Based on 2424 observations of 1266 individuals and representing results from logistic regression models regressing young adult outcome variables on lagged despair scores. All models included the following covariates: sex, race/ethnicity, educational level, poverty status, and lagged value of the outcome variable.

^b^ Significant at *P* < .05.

### Moderation by Educational Level, Race/Ethnicity, Sex, and Poverty

A series of models tested whether observed associations between despair and later outcomes were moderated by sociodemographic factors. For example, does this association differ between male and female participants or by educational level? Moderation was tested with an interaction term between despair and the sociodemographic factor. eTable 5 in the Supplement shows the result of the moderation tests for each of the variables. Although the results of 4 of the 20 interaction tests were significant, there was no consistent pattern of moderation, either in terms of outcomes or moderators. Follow-up analyses were conducted for each moderator group separately to facilitate future systematic reviews or meta-analyses (eTables 6, 7, 8, and 9 in the Supplement).

## Discussion

Despair has been postulated as a cause of the recent increase in premature mortality associated in part with suicide and alcohol- and drug-related deaths. This study tested the longitudinal associations between despair and suicidal thoughts and behavior and substance misuse in young adulthood. Despair scores were highest among those with lower educational level and income status and among African American participants. Despair scores were longitudinally associated with increased levels of suicidal thoughts and behavior, illicit drug use, illicit drug use disorders, and opioid use over time but not alcohol use disorder. These associations were observed even after accounting for depression status. The observed despair-outcome associations were stronger in models accounting for long-term measures of despair. This study provides an empirical basis for despair as longitudinally associated with suicidality and illicit substance use.^20^

How do our findings inform our understanding of diseases and deaths of despair? Despair was both concurrently associated with the outcomes and preceded them. These associations persisted in models accounting for a number of other sociodemographic factors strongly associated with these diseases as well as depressive disorders. Our findings suggest a 1.4 to 1.7 times prospective increase in the odds of each outcome for each additional indicator of despair reported. (In models using a dichotomized despair variable, the ORs were around 2 to 3.) The associations were stronger when accounting for long-term despair exposure extending back to childhood. In contrast, none of the diseases were associated with increased levels of despair in longitudinal models. These results suggest that the pathway from despair to diseases of despair is unidirectional.

In this analysis, which tested a limited number of outcomes, the associations of despair were specific to suicidal behavior, illicit drug use, illicit drug use disorder, and opioid use. These findings are consistent with evidence from other studies that suggest that suicidal behavior and drug use are a result of diminished valuing of oneself and one’s future.^21,22,23^ Despair was not associated with alcohol use disorder, however. Alcohol use disorder was equally common among those with the lowest levels of despair as in those with the highest levels of despair. This study does not support alcohol use disorder as a disease of despair.

As expected, despair was more common among those with low educational level or limited financial resources. At the same time, the associations of despair with outcomes were neither stronger nor weaker in specific sociodemographic groups. In other words, the relative associations of despair with outcomes did not differ systematically between groups. This finding is consistent with recent reports that the newest premature mortality trends appear to be mostly universal across US racial/ethnic groups.^2^ Accordingly, public policy strategies should focus on population-level interventions rather than targeting specific subgroups to reduce despair.

Despair is a new construct, but the associated *DSM*-based depressive disorders have been studied intensely for the past 50 years or more.^24,25,26,27^ Is despair useful or is it simply a new way of framing an old condition? In this study, many of those who met the criteria for a depressive disorder also displayed despair. However, the association was not reciprocal: most individuals reporting despair did not meet the criteria for a depressive disorder. It might be expected that individuals with both despair and depressive disorder would be at risk for suicidal thoughts and behavior and substance misuse, but this was not the case. Depression is a complex medical disorder seen in comparatively few individuals, whereas despair is a cognitive status experienced by many individuals that may reflect the social and economic deterioration of many US communities. In this study, despair scores had an independent utility in understanding risk for these high-priority diseases.

This literature has understandably focused on the extreme outcomes of reduced life expectancy and premature mortality. Our findings suggest that despair is common in young adulthood and is associated with destructive behaviors that may or may not lead to mortality. The focus on mortality alone may miss a broader association of despair with impairment and reduced quality of life. In the language of the Global Burden of Disease study, the public health outcome of the disability-adjusted life-years associated with despair may far outstrip the association with mortality itself.^26^ Future studies should not only focus on outcomes associated with mortality but also explore how despair is associated with important areas of life functioning, such as financial and social functioning and physical health.

### Limitations

This study has some limitations. The sample is not representative of the US population. Measures of despair were obtained from parent and child report to 16 years of age and only from self-report thereafter. The despair scale was created for the present study post hoc using items available from the structured interview. The focus of each observation was on despair indicators within the prior 3 months only, thus allowing it to miss despair in the intervening periods. Although participation rates were high at young adult assessments (>80%), the results may be affected by missing observations. Also, we studied common precursors of deaths of despair rather than mortality per se because the latter was not possible with our young adult sample. This study did not clarify whether opioid use was prescription or nonprescription, which may lead to some misclassification. More important, the opioid use results closely mirror the results for other illicit drug use, suggesting that such misclassification is limited. Finally, the study is a cohort study and unable to test causal associations between despair and suicidality or substance misuse.

## Conclusions

In the play *Caesar and Cleopatra*, George Bernard Shaw observes “he who has not hoped can never despair.”^27^^(p189)^ Despair may be a risk factor for diseases, but it is also the end point of a process in which hope is lost. The public health successes of the 20th century have consistently allowed children to live longer than their parents. It is precisely because this recent pattern deviates from a century of progress that it has captured the attention of the public and the scientific community. This study takes a step toward understanding how a psychological state could derail the seemingly inexorable progress of modern medicine. However, this study is only 1 step, and additional work is needed to understand the origins of despair and premature mortality within individuals, families, and communities and how we can intervene to recover hope and forestall further morbidity and mortality.

## Notes

Supplement. **eTable 1.** Individual Indicators of Cognitive Despair and Their Definitions **eTable 2.** Definitions of Different Types of Suicidal Thoughts and Behaviors **eTable 3.** Associations of Lagged Despair Scores With Young Adult (age 25, 30) Outcomes **eTable 4.** Models Predicting Young Adult Outcomes (ages 25 and 30) Comparing Different Levels of Lagged Despair (0 vs 1, 2, 3) **eTable 5.** Lagged Longitudinal Models Testing Interactions Between Despair and Each of, Sex, Race/Ethnicity, Poverty, and Educational Attainment in the Prediction of Each Outcome **eTable 6.** Lagged Models Predicting Young Adult Outcomes Separately by Poverty Status **eTable 7.** Lagged Models Predicting Young Adult Outcomes Separately by Educational Attainment **eTable 8.** Lagged Models Predicting Young Adult Outcomes Separately by Race/Ethnicity **eTable 9.** Lagged Models Predicting Young Adult Outcomes Separately by Sex **eFigure.** Ascertainment of the Original Great Smoky Mountains Study Sample
