# Supplementary material for: Associations of Despair With Suicidality and Substance Misuse Among Young Adults
Source: JAMA Netw Open. 2020 Jun 23;3(6):e208627. doi: 10.1001/jamanetworkopen.2020.8627 (PMC7312388; doi:10.1001/jamanetworkopen.2020.8627)
Supplement: Supplement. — eTable 1. Individual Indicators of Cognitive Despair and Their Definitions eTable 2. Definitions of Different Types of Suicidal Thoughts and Behaviors eTable 3. Associations of Lagged Despair Scores With Young Adult (age 25, 30) Outcomes eTable 4. Models Predicting Young Adult Outcomes (ages 25 and 30) Comparing Different Levels of Lagged Despair (0 vs 1, 2, 3) eTable 5. Lagged Longitudinal Models Testing Interactions Between Despair and Each of, Sex, Race/Ethnicity, Poverty, and Educational Attainment in the Prediction of Each Outcome eTable 6. Lagged Models Predicting Young Adult Outcomes Separately by Poverty Status eTable 7. Lagged Models Predicting Young Adult Outcomes Separately by Educational Attainment eTable 8. Lagged Models Predicting Young Adult Outcomes Separately by Race/Ethnicity eTable 9. Lagged Models Predicting Young Adult Outcomes Separately by Sex eFigure. Ascertainment of the Original Great Smoky Mountains Study Sample [file jamanetwopen-3-e208627-s001.pdf]

Supplementary Online Content

Copeland WE, Gaydosh L, Hill SN, et al. Associations of despair with suicidality and substance misuse among young adults. *JAMA Netw Open.* 2020;3(6):e208627. doi:10.1001/jamanetworkopen.2020.8627

eTable 1. Individual Indicators of Cognitive Despair and Their Definitions
eTable 2. Definitions of Different Types of Suicidal Thoughts and Behaviors
eTable 3. Associations of Lagged Despair Scores With Young Adult (age 25, 30) Outcomes
eTable 4. Models Predicting Young Adult Outcomes (ages 25 and 30) Comparing Different Levels of Lagged Despair (0 vs 1, 2, 3)
eTable 5. Lagged Longitudinal Models Testing Interactions Between Despair and Each of, Sex, Race/Ethnicity, Poverty, and Educational Attainment in the Prediction of Each Outcome
eTable 6. Lagged Models Predicting Young Adult Outcomes Separately by Poverty Status
eTable 7. Lagged Models Predicting Young Adult Outcomes Separately by Educational Attainment
eTable 8. Lagged Models Predicting Young Adult Outcomes Separately by Race/Ethnicity
eTable 9. Lagged Models Predicting Young Adult Outcomes Separately by Sex
eFigure. Ascertainment of the Original Great Smoky Mountains Study Sample

This supplementary material has been provided by the authors to give readers additional information about their work.

© 2020 Copeland WE et al. *JAMA Network Open.*

**eTable 1.** Individual Indicators of Cognitive Despair and Their Definitions		
Item	Definition	Relation to *DSM* disorders
Loneliness	Feeling of being alone and/or friendless.	No disorder
Hopelessness	Bleak, negative, pessimistic view of future and little hope that his/her situation will improve.	Dysthymic disorder
Helplessness	Feels there is little or nothing s/he can do to improve his/her situation	No disorder
Low self-esteem	Unjustified feeling of inferiority to others or severe hostility against him/herself.	Dysthymic disorder
Frequent worries	Painful, unpleasant, or uncomfortable thoughts about future, past behavior, competence, calamities, money, appearance that cannot be stopped voluntarily	Generalized anxiety disorder
Feels sorry for self	A feeling that life or people have been unfairly unpleasant or troubling.	No disorder
Feels unloved	Generalized feeling of being unloved and uncared for.	Major depressive disorder

© 2020 Copeland WE et al. *JAMA Network Open.*

**eTable 2.** Definitions of Different Types of Suicidal Thoughts and Behaviors	
Construct	Definition
Passive ideation	Thoughts about wanting to be dead or death. Include thoughts about not being able to go on any longer and life not being worth living. To code, thoughts must be intrusive into at least two activities.
Active ideation	Thoughts specifically about killing oneself, by whatever means, with some intention to carry them out. Do not include suicidal plans
Suicidal Plans	Suicidal thoughts that contain plans of a suicidal act and some intent to carry them out. If suicidal attempt has been made, determine whether a plan was present prior to the attempt.
Suicide Attempt	Episodes of deliberately self-harmful behavior involving some intention to die at the time of the attempt. Rate here, no matter how unlikely the attempt was to cause death, so long as the child's intention was to die. If parent unsure about intention to die, code if the parent can describe a clear self-harmful event.

© 2020 Copeland WE et al. *JAMA Network Open.*

**eTable 3.** Associations of Lagged Despair Scores With Young Adult (age 25, 30) Outcomes						
		Despair scores				
	Total	0	1	2	3+	
	n (%)	n (%)	n (%)	n (%)	n (%)	*P* Value
**Total**	2424 (100)	1937 (80.5)	275 (12.0)	94 (4.1)	107 (3.5)	
**Suicide**						
Yes	160 (5.9)	102 (4.2)	23 (9.7)	16 (15.3)	19 (24.1)	--
No	2253 (94.1)	1907 (95.8)	247 (90.3)	57 (84.7)	42 (75.9)	**0.001**
**Alcohol disorder**						
Yes	132 (7.6)	109 (7.5)	11 (5.8)	7 (3.9)	5 (3.6)	--
No	2292 (92.4)	1910 (92.5)	260 (94.2)	66 (96.1)	56 (96.4)	0.13
**Illicit drug use**						
Yes	137(4.7)	102 (3.8)	17 (3.7)	6 (9.6)	12 (27.1)	**--**
No	2247 (95.3)	1885 (96.2)	248 (96.3)	66 (90.4)	48 (72.9)	**<0.001**
**Illicit drug disorder**						
Yes	88 (2.9)	68 (2.6)	10 (1.5)	4 (2.7)	6 (9.9)	**--**
No	2336 (97.1)	1951 (97.4)	261 (98.5)	69 (97.3)	55 (90.1)	**0.68**
**Opioid Use**						
Yes	55 (2.2)	41 (1.7)	6 (1.0)	2 (6.8)	6 (16.5)	**--**
No	2328 (97.8)	1945 (98.3)	259 (98.9)	70 (93.2)	54 (83.5)	**0.002**

Table is based upon 2424 observations of N=1266 individuals. All percentages are weighted and n is unweighted. Despair score is treated as a count variable. Covariates included sex, race/ethnicity, educational status, and poverty. Bolded values are significant at *p* <0.05.

© 2020 Copeland WE et al. *JAMA Network Open.*

**eTable 4.** Models Predicting Young Adult Outcomes (ages 25 and 30) Comparing Different Levels of Lagged Despair (0 vs 1, 2, 3)							
		0 vs 1		0 vs. 2		0 vs. 3	
Outcome	Predictors	OR	95% CI	OR	95% CI	OR	95% CI
Suicidality	Despair	1.7	0.8-3.8	1.6	0.3-7.3	3.4	1.2-9.9
Illicit drug use	Despair	0.9	0.3-2.3	3.0	0.7-12.2	5.6	1.7-18.5
Opioid Use	Despair	0.7	0.2-2.1	4.3	0.9-21.2	7.2	1.5-35.6

Table is based on 2424 observations of 1266 individuals. Covariates included sex, race/ethnicity, educational status, poverty. Bolded values are significant at *p* <0.05. OR= odds ratio; CI = 95% confidence intervals.

© 2020 Copeland WE et al. *JAMA Network Open.*

eTable 5. Lagged Longitudinal Models Testing Interactions Between Despair and Each of, Sex, Race/Ethnicity, Poverty, and Educational Attainment in the Prediction of Each Outcome

	Sex	Race/ethnicity	Poverty	Educational Attainment
	P Value	*P* Value	*P* Value	*P* Value
Outcome				
Suicide	**0.03**	0.53	**0.01**	0.44
Alcohol disorder	0.75	0.11	0.84	0.08
Illicit drug use	0.08	**0.03**	0.71	0.07
Illicit drug disorder	0.99	0.36	0.90	**0.008**
Opioid Use	0.09	0.55	0.62	0.13

Bolded values are significant at $p < 0.05$.

© 2020 Copeland WE et al. *JAMA Network Open.*

**eTable 6.** Lagged Models Predicting Young Adult Outcomes Separately by Poverty Status						
	Impoverished			Not impoverished		
Outcome	OR	95% CI	p			
Suicide	2.2	1.3-3.5	0.78	0.8	0.5-1.3	0.41
Alcohol disorder	0.7	0.4-1.3	0.28	0.8	0.5-1.4	0.51
Illicit drug use	1.7	1.0-3.0	0.06	1.7	0.9-3.2	0.14
Illicit drug disorder	0.8	0.4-1.6	0.52	1.3	0.8-2.0	0.31

Poverty status was defined based upon Federal definitions incorporating household size and income.

© 2020 Copeland WE et al. *JAMA Network Open.*

**eTable 7.** Lagged Models Predicting Young Adult Outcomes Separately by Educational Attainment						
	Low attainment			Higher attainment		
Outcome	OR	95% CI	p			
Suicide	1.5	0.6-3.9	0.38	**1.6**	**1.0-2.4**	**0.03**
Alcohol disorder	0.5	0.3-1.1	0.07	1.1	0.7-1.7	0.85
Illicit drug use	1.2	0.6-2.3	0.65	**2.6**	**1.7-4.2**	**<0.001**
Illicit drug disorder	0.6	0.2-1.6	0.30	**1.9**	**1.3-2.8**	**<0.001**

High educational attainment was defined as finishing at least some college; low attainment was defined failing to complete high school or obtaining a high school degree only.

© 2020 Copeland WE et al. *JAMA Network Open.*

**eTable 8.** Lagged Models Predicting Young Adult Outcomes Separately by Race/Ethnicity						
	White			Native American		
Outcome	OR	95% CI	p			
Suicide	1.3	0.9-1.9	0.12	--	--	--
Alcohol disorder	0.8	0.5-1.2	0.25	1.6	0.9-2.9	0.10
Illicit drug use	**1.6**	**1.1-2.4**	**0.02**	0.7	0.4-1.5	0.38
Illicit drug disorder	1.1	0.6-1.9	0.87	0.8	0.5-1.3	0.81

Only the comparison between white participants and Native American participants was tested. This table excluded African Americans (n=79) as that group is too small for stand-along comparisons.

© 2020 Copeland WE et al. *JAMA Network Open.*

**eTable 9.** Lagged Models Predicting Young Adult Outcomes Separately by Sex						
	Male			Female		
Outcome	OR	95% CI	p			
Suicide	**2.3**	**1.4-3.8**	**<0.001**	1.1	0.7-1.5	0.79
Alcohol disorder	0.7	0.5-1.2	0.22	0.7	0.3-1.5	0.30
Illicit drug use	**2.2**	**1.4-3.3**	**<0.001**	1.2	0.7-2.1	0.53
Illicit drug disorder	1.1	0.5-2.7	0.81	1.0	0.5-1.9	0.95

© 2020 Copeland WE et al. *JAMA Network Open.*

eFigure. Ascertainment of the Original Great Smoky Mountains Study Sample

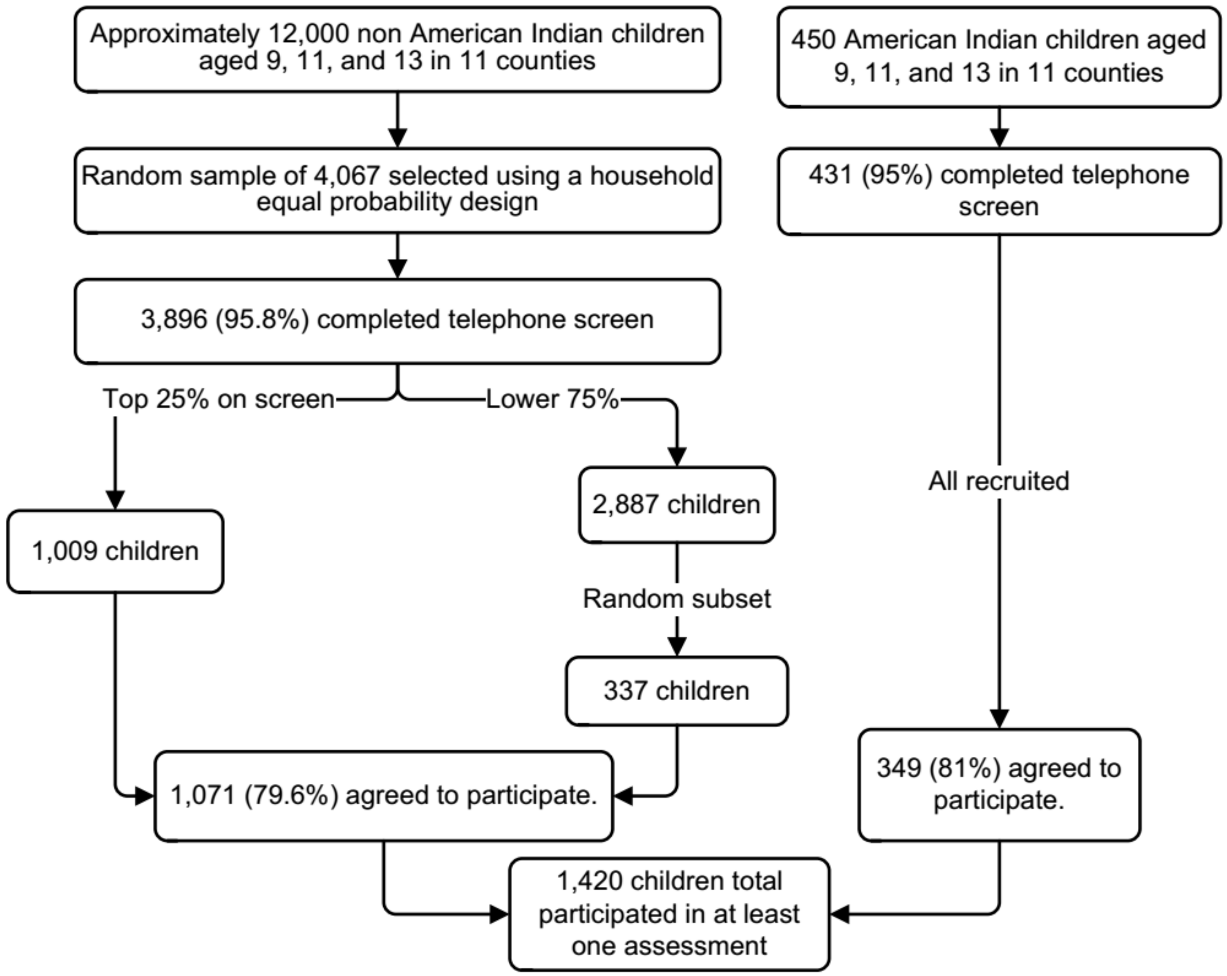

Telephone screen assessed for psychopathology risk.

© 2020 Copeland WE et al. *JAMA Network Open.*
